# The aqueous extract of brucea javanica reduces tumorigenicity of human lung cancer tumorspheres

**DOI:** 10.20517/cdr.2021.58

**Published:** 2021-08-13

**Authors:** Seung-Hun Kim, Po-Wei Fan, Chang-Heng Hsieh, Hsuan-Yuan Lin, Wen-Hsin Wang, Ming-Chung Lee, Kang Fang

**Affiliations:** ^1^School of Life Science, Department of Life Science, National Taiwan Normal University, Taipei 116, Taiwan.; ^2^Brion Research Institute of Taiwan, New Taipei City 231, Taiwan.

**Keywords:** *Brucea javanica*, cancer stem cells, drug resistance, epidermal growth factor receptor, lung cancer, apoptosis

## Abstract

**Aim:** Therapy to overcome drug resistance by modulating epidermal growth factor receptor (EGFR) is a viable approach to suppress the proliferation of human non-small cell lung cancer (NSCLC) cells. A previous study demonstrated that the seeds of an aqueous *Brucea javanica *(BJ) (L.) Merr (Simaroubaceae) extract containing quassinoid mixtures effectively inhibited the growth and alleviated tumorigenesis in H1975 cells of NSCLC by targeting T790M/L858R EGFR. This study aimed to further determine whether the aqueous BJ extract affects the enriched H1975 spheroids in suspension culture and mouse xenograft tumor models.

**Methods:** The spheroids of NSCLC adenocarcinoma H1975 cells were enriched in a serum-free media. The growth rate of sphere propagation by aqueous BJ extract was determined in suspended culture and in colony-formation assay. BJ extract was fed orally to nude mice bearing xenograft tumors. The resected tumors were analyzed by hematoxylin and eosin staining, terminal deoxynucleotidyl transferase-mediated dUTP nick-end labeling assay, and proliferating cell nuclear antigen assessment. Various markers were used to determine the pluripotency of tumors from mice treated with different concentrations of BJ extract.

**Results:** BJ extract was demonstrated to be effective against the propagation of the enriched spheroids. In animal models, oral administration of the aqueous BJ extract reduced spheroid tumorigenicity. The alleviated growth of the established xenograft tumors can be attributed to the reduced drug resistance and induced apoptosis without distinct adverse effects. More evidence supports activated apoptotic death attenuated spheroid stemness of tumors.

**Conclusion:** As an effective treatment regime to assuage lung cancer, the indigenous BJ extract promises to obliterate drug resistance and the growth of cancer stem cell tumors from NSCLC cells harboring T790M/L858R EGFR.

## INTRODUCTION

Finding strategies against drug-resistant cancer stem-like cells (CSCs) to reinforce the conventional cancer treatment is a challenging task^[1]^. The minor cell subpopulations within the CSCs are closely related to the onset of malicious non-small cell lung cancer (NSCLC) tumors. The minor and slow-dividing CSCs refractive to various treatments account for the recurrence of lung tumors^[2]^. The enriched progenitor clusters propagate in the environment of serum-free medium with supplementation of growth factors. The enriched spheroid fractions commonly referred to as CSCs can be suspended and propagated as tumorspheres^[2,3]^.

Epidermal growth factor receptor (EGFR) signal activation in NSCLC cells contributes to tumor growth and impedance to apoptosis. Measures to subdue resistance to EGFR inhibitors turned out to be an immense obstacle in treating NSCLC patients^[4]^. The biomarker exhibiting mutated EGFR at L858R in NSCLC patient is a prerequisite for the effectiveness of tyrosine kinase inhibitors (TKIs) such as gefitinib^[5]^. However, the progressive *EGFR* mutation at amino acid 790 (T790M) during TKI therapy increasingly blocked steric binding of the drug followed by severe therapy resistance^[6,7]^. This complication prompted more development in therapy by targeting the durable stem-like cell populations that are resistant to TKI because of the evolved double mutant EGFR^[8,9]^. More evidence supports that, by targeting populations of CSC comprising aberrant EGFR, the growth of NSCLC can be suppressed and, finally, eradicated^[3]^. Thus, to develop new efficient therapeutic drugs, improvements for suppressing NSCLC cells of mutated EGFR are crucial in attenuating growth of malignant tumors^[7,10]^.

Indigenous plants are frequently known to revert multi-drug resistance by activating cell death during cancer treatment^[11]^. One of the commonly accepted herb medicines is from the fruits of *Brucea javanica *(BJ) (L.) Merr, a plant species of the evergreen shrub family Simaroubaceae widely distributed in Southeast Asia and northern Australia^[12]^. For years, the harvested fruits have been widely accepted as a remedy for diseases in East Asia and a traditional medicine^[13]^. The BJ fruits were proved potent against pancreatic cancer growth^[14]^. The ethyl acetate extract of the seeds can cure patients with inflammatory diseases^[15]^ and allergy^[9]^. The fruits were also efficacious in suppressing the growth of various types of cancer^[14,16,17]^. A combination of chemotherapy drugs with javanica oil emulsion is used to treat gastric cancer^[18]^ and lung adenocarcinoma^[19]^. Other reports indicate that aqueous BJ extract inhibited the growth of liver cancer cells and the enriched spheroids^[20]^. 

The malignant tumorspheres are a minor population of cells endowed with CSC characteristics. The malicious tumor-initiating cells tend to recur and invade, and they are metastatic and resistant to therapy as neoplasm progresses^[21]^. The inherent self-renewal abilities in tumorspheres lead to resistance to environmental affronts and drug intervention^[8]^. BJ extract was demonstrated effective against tumor development of NSCLC cells with EGFR carrying T790M/L858R^[22]^. To demonstrate more translational values, it is imperative to learn if the aqueous BJ extract prevents the propagation of three-dimensional spheres of lung cancer cells. To serve this purpose, cells were enriched to form spheroids, in which the development of intrinsic stemness and epithelial-mesenchymal transition (EMT) features of CSCs were assessed. The study began with determining the sensitivity of the aqueous extract against H1975 cell spheroids followed by animal model evaluation. The aim was to determine whether BJ is effective against minor but malicious lung cancer tumorspheres.

## METHODS

### Liquid chromatography and mass spectrometry (LC/MS) fingerprint analysis

The LC/MS method was used to identify the major bioactive substances, which included a Shimadzu LC-20AD UFLC system linked to an LC/MS-8040 triple quadrupole mass spectrometer. The running condition included gradient elution with a mixture of mobile phases A (0.1% formic acid and 1 g/L solution of ammonium acetate in water) and B (0.1% formic acid and 1 g/L solution of ammonium acetate in methanol) at Minutes 0-40 with the ratio of 100% to 70% A and 0% to 30% B; at Minutes 40-70 with the ratio of 70% to 0% A and 30% to 100% B; at Minutes 70-70.1 with the ratio of 0% to 100% A and 100% to 0% B; and at Minutes 70.1-80 with the ratio of 100% A and 0% B. The flow rate was set at 0.4 mL/min and the column temperature kept at 40 °C. The injection volume was adjusted to 30 μL, and the analytical column used a Shimadzu Shim-pack XR-ODS II column (2.2 μm, 2 mm × 100 mm, Shimadzu). Dual ion modes [electrospray ionization ESI (+) and ESI (-)] were used in MS detection and the transmission of [M+H]^+ ^and [M-H]^- ^was set as the optimum condition. The MS detection was arranged as a full scan range (400-800 amu). The interface voltages were set at 4.5 kV for ESI (+) and -3.5 kV for ESI (-). With nitrogen as the nebulizing and drying gas, the flow was set at 3.0 and 10 L/min, respectively. Argon as the collision-induced dissociation gas was kept at 230 kPa. Desolvation line temperature was set at 150 °C, and the heat block temperature was maintained at 400 °C.

### Cell culture

Human NSCLC cells H1975 [CD133 rich, two mutations in EGFR (T790M /L858R)] were acquired from American Type Culture Collection (Manassas, VA) and cultured in 75 cm^2^ tissue culture flasks. The cells were cultured in Dulbecco’s Modified Eagle Medium (DMEM) containing 10% fetal bovine serum (FBS) (Thermo Fisher Scientific, Waltham, MA, USA), 100 unit/mL penicillin, and 100 μg/mL streptomycin and maintained at 37 °C in the environment of humidified incubator with 5% CO_2_.

### Chemicals and reagents

Sun Ten Pharmaceutical (Taichung, Taiwan) provided the aqueous extract of the whole *BJ* plant for experiments. Briefly, 100 g of the collected materials were extracted with sterile water as previously published^[20]^. The samples of the final concentrates were adjusted to make a concentration of 1 g/mL and stored at 25 °C. The chemicals 4’,6-diamidino-2-phenylindole (DAPI), crystal violet, penicillin-streptomycin, glutamine, trypsin-EDTA, and DMEM medium were obtained from Thermo Fisher Scientific (Waltham, MA, USA).

### Enrichment of spheroids

To enrich undifferentiated tumorspheres, NSCLC adenocarcinoma H1975 cells were cultured with serum-free DMEM-F12 medium (Thermo Fisher Scientific) in ultra-low attachment tissue culture plates to maintain cell suspension (Corning, Corning, NY). The culture condition included N-2 Plus Media Supplement (R&D Systems, Minneapolis, MN), B-27 Supplement (Thermo Fisher Scientific), 20 ng/mL of epidermal growth factor (Pepro Tech, Rocky Hill, NJ), and 10 ng/mL of basic fibroblast growth factor (bFGF) (Pepro Tech). The floating sphere cultures were expanded by mechanical dissociation, followed by re-plating single cells in a fresh medium. For maintenance, one-third of the serum-free media was replaced every 3 days. The collected spheroids were subjected to subsequent experiments in non-adherent culture conditions. Spheroid diameters were measured using Image-Pro Plus 6.3 (Media Cybernetics, Bethesda, MD). 

### Colony-formation assay

The growth of spheroids was determined by a colony formation assay. A total of 1 × 10^6^ cells per well in 75 cm^2^ ultra-low attachment tissue culture plates were raised in growth factor-supplemented medium for 7 days to allow the spheroids to reach a size of more than 50 μm in diameter. The spheres were then treated with different concentrations of BJ extract for 12 h, and the mixtures were cultured in soft agar with 0.5% agarose on top and 0.3% agar on bottom layers. After 28 days, the plates were stained with 0.002% (w/v) crystal violet and incubated at 37 °C for 30 min. Following removal of the dye solution, colonies of more than 50 cells were counted as positive. Three individual experiments were conducted for each condition.

### Western blot analysis of spheroid cell lysates

The spheroids were formed by plating 1 × 10^6^ cells per well in serum-free media with supplementation of growth factors and treated with various concentrations of aqueous extractions of BJ (0, 5, 10, and 15 mg/mL) for 12 h. The harvested cell pellets were lysed and the protein concentrations determined using a Bio-Rad protein assay kit for (Hercules, CA, USA) before being resolved by electrophoresis and transferred to nitrocellulose membrane. The blots were blocked with 5% non-fat milk and incubated with 1:2000 dilutions of primary antibodies, including anti-pAkt (GTX128414); anti-PARP (GTX112864); anti-EGFR (GTX121919); anti-pEGFR (GTX61507), anti-ABCG2 (GTX100437), anti-Nanog (GTX100863), anti-CD133 (GTX100567), anti-Sox2 (GTX627405), and anti-ALDH1A1 (GTX123973), from GeneTex. Membranes were then incubated with 0.3 µg/mL of peroxidase-conjugate anti-mouse or anti-rabbit IgG (Thermo Fisher Scientific) and detected with enhanced chemiluminescence substrate (Thermo Fisher Scientific). The loading control was incubated with anti-GAPDH antibody (GTX100118, GeneTex). The signals were visualized with enhanced LAS-4000 (FUJIFILM) apparatus and the band intensities of images analyzed using ImageJ software.

### Bromodeoxyuridine incorporation of spheroids

The assays followed the published procedures^[20]^. The assays to determine the proliferation of spheres used bromodeoxyuridine (BrdU) Labeling and Detection Kit II (Roche, Mannheim, Germany). The enriched spheroids following treatment with BJ extract for 48 h were incubated in media containing 10 μM BrdU for 1 h. Fixatives of 3:7 mixture of 50 mM glycine solution (pH 2.0) and ethanol were added followed by incubation overnight at -20 °C. The slides were washed with phosphate-buffered saline (PBS) and incubated at 37 °C in a solution containing diluted anti-BrdU antibody (1:1000 dilution, GeneTex) for 30 min. The cells were suspended in a mixture of 1% bovine serum albumin containing Alexa Fluor 488-conjugated secondary antibody (1:250 dilution) for 2 h in darkness. An inverted fluorescence microscope was used to record the fluorescent images. All colored images were converted to black and white by Photoshop software before being quantitated with Multi Gauge software (version 2.1, FUJIFILM). The green fluorescence intensities at each concentration as obtained were compared with that of the water control as the BrdU intensity ratio. Three independent experiments were carried out.

### TUNEL assay

The apoptotic death as evaluated by terminal deoxynucleotidyl transferase-mediated dUTP nick-end labeling (TUNEL) staining of cultured spheroids and resected tissues was performed following the procedures as described previously^[20]^. Cells treated with BJ extract for 12 h underwent permeabilization and were blocked before being incubated in TUNEL reaction mixtures. The colored images were then converted to black and white by Photoshop software and quantitated with Multi Gauge software (version 2.1, FUJIFILM). The positively stained cells were calculated and converted into percentages. Three independent experiments were carried out.

### Xenograft tumor evaluation in mice

The subcutaneous xenograft animal model was established for tumorigenesis evaluation. The three- or four-week-old female *nu/nu* mice were obtained from the National Applied Research Laboratories (Taipei, Taiwan). The animals were housed under aseptic and ventilated conditions free of pathogens with 12 h light and dark cycle. Before treatment, mice were acclimated for 7 days. The animal protocols were approved by the Animal Committee of the Institution. 

A total of 8 × 10^4 ^of H1975 spheroid cells were suspended in 0.2 mL mixture of PBS and Matrixgel™ Basement Membrane Matrix (BD Biosciences) (1/1, v/v) and inoculated subcutaneously into the dorsal area of nude mice. Each group consisted of four mice. When the xenograft tumors reached 50-100 mm^3 ^in size following sphere implantation for 14 days, the mice were fed orally with 2 and 4 g of BJ/kg in the treatment group or an equal volume of water for controls every day for 6 consecutive days. The health conditions and body weights of the animals were closely monitored. The tumor size at each time point was determined before gavage feeding. The dimensions of the xenografts (longitudinal length and transverse width) were measured using an electronic digital caliper and the measurements converted to the xenograft volume (π/6  ×  width^2 ^×  length). Sixteen days after final feeding, the mice were sacrificed under CO_2_ and the collected tumor samples resected for further analysis. One-way ANOVA tests were used for statistical comparisons between different groups.

Tumors were removed from the mice after being sacrificed and fixed with 5% (w/v) freshly prepared formaldehyde overnight at room temperature. For the tissue section, the fixed tumors were sliced into 0.5-1 cm thick slabs and passed in the order of 10%, 20%, and 30% (w/v, PBS) sucrose solutions. The slabs were frozen, equilibrated in a cryostat at -20 °C, and then resected by a cryostat microtome (LEICA). For immunostaining, tumor sections were incubated overnight in 5% FBS (pH 7.4) diluted in PBS that contained 0.2% Triton X-100. The tumor sections were washed in PBS and incubated with various primary and dye-conjugated secondary antibodies before being visualized with confocal microscopy. Tissue sections were incubated with 3 µg/mL of TRITC- or FITC-conjugated anti-rabbit IgG (Thermo Fisher Scientific) for 1 h and examined by confocal microscopy.

### Statistical analysis

All data represent means ± standard deviation of three individual experiments. The statistical analysis using Student’s *t*-test was performed using GraphPad Prism 5.00 for Windows (GraphPad, San Diego, CA, USA). Statistical differences were considered significant with *P *< 0.05.

## RESULTS

### LC/MS composition analysis of aqueous BJ extract

**Figure 1:**
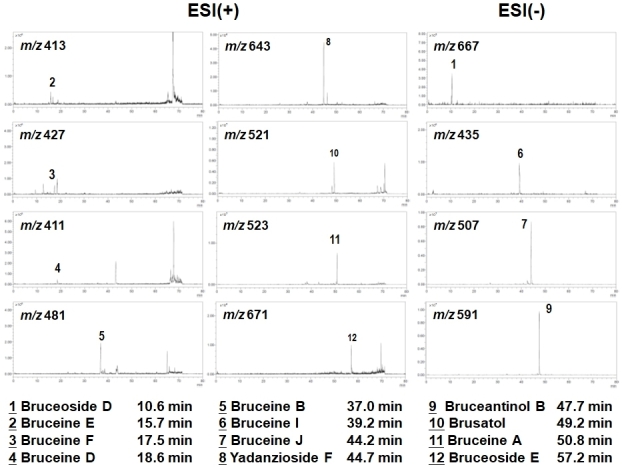
LC/MS fingerprint analysis. Chromatographic fingerprint analysis was conducted using LC/MS analysis. In total, 12 bioactive marker substances from ingredients of the BJ were identified within 80 min under the selected LC/MS condition. The peaks at the specific retention time were: bruceoside D (Peak 1, 10.6 min), bruceine E (Peak 2, 15.7 min), bruceine F (Peak 3, 17.5 min), bruceine D (Peak 4, 18.6 min), bruceine b (Peak 5, 37.0 min), bruceine I (Peak 6, 39.2 min), bruceine J (Peak 7, 44.2 min), yadanzioside F (Peak 8, 44.7 min), bruceantinol B (Peak 9, 47.7 min), brusatol (Peak 10, 49.2 min), bruceine A (Peak 11, 50.8 min), bruceoside E (Peak 12, 57.2 min).

The obtained typical LC/MS chromatographic fingerprint profile of aqueous BJ extract showed twelve major components [Figure 1]. The resolved peaks with retention times of less than 40 min were identified as: (1) bruceoside D (PubChem CID: 10484578); (2) bruceine E (PubChem CID: 122785); (3) bruceine F (17.5 min); (4) bruceine D (PubChem CID: 441788); (5) bruceine B (PubChem CID: 161496); and (6) bruceine I (PubChem CID: 196839). The distinct peaks above 40 min included: (7) bruceine J (PubChem CID: 23656476); (8) yadanzioside F (PubChem CID: 3000798); (9) bruceantinol B (PubChem CID: 23656477); (10) brusatol (PubChem CID: 73432); (11) bruceine A (PubChem CID: 160006); and (12) bruceoside E (PubChem CID: 3000803).

### BJ extract reduced the growth of cell spheroids

**Figure 2:**
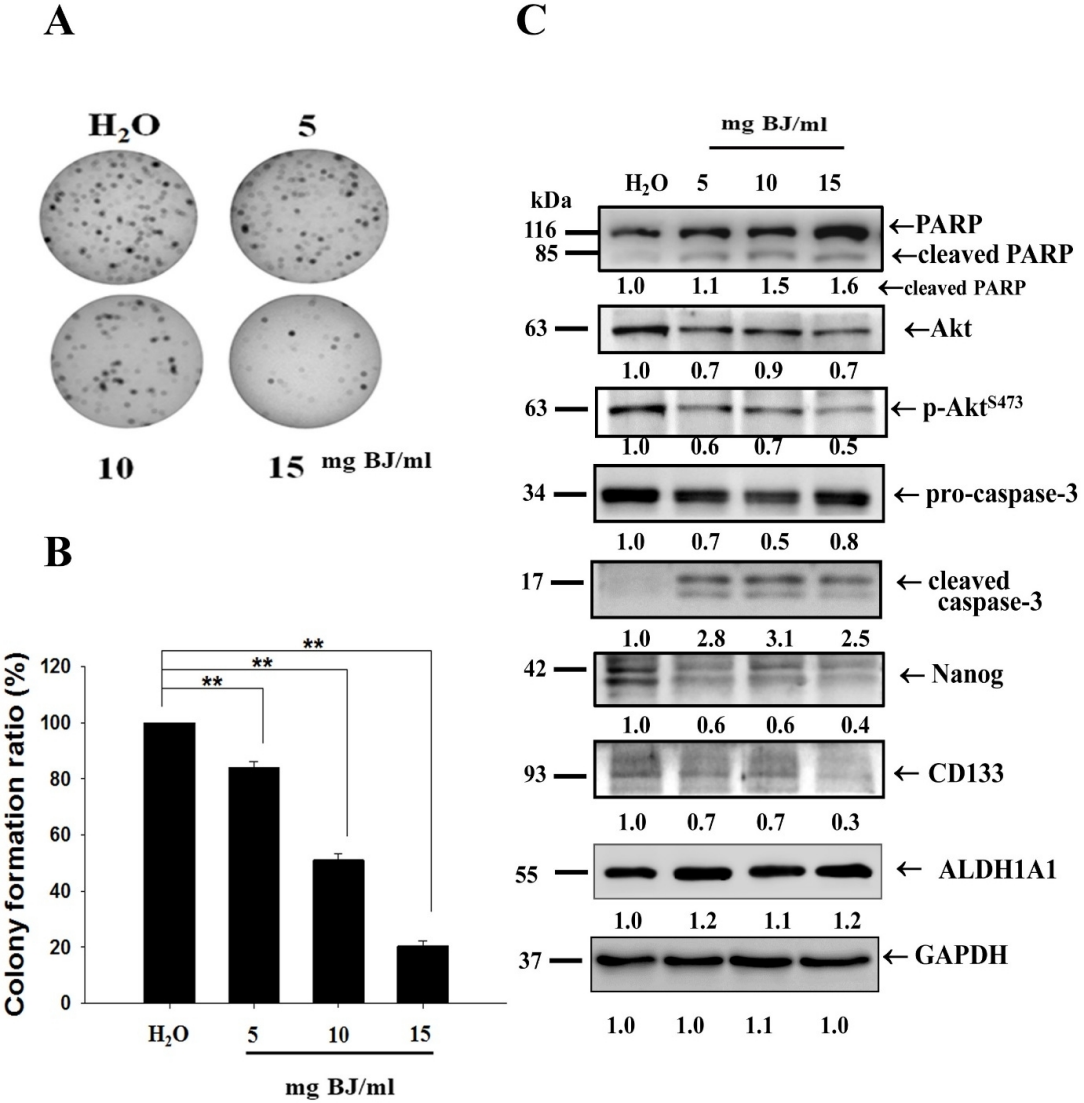
BJ affected the growth of multicellular H1975 spheroids. (A) Soft agar colony-forming assay. The multicellular spheroids treated with 5, 10, and 15 mg/mL of BJ extract were grown in soft agar for 28 days and the plates were stained with crystal violet, as described in “Methods”. (B) Statistical analysis. The number of positive soft agar colonies of H1975 spheroids under each concentration was counted and plotted. Colonies containing more than 50 cells were counted as positive. **P < 0.01 indicates a significant difference between BJ treatment groups and water control from three independent experiments. (C) BJ extract reduced H1975 spheroid stemness markers. Cell lysates from H1975 spheroids as treated with BJ extract for 12 h were subjected to Western blot analysis. The blots were incubated with primary antibodies and analyzed for expressions of apoptosis and stemness markers with GAPDH as loading control, as described in “Methods”. The numbers underneath indicate the relative intensities compared with water treatment.

A previous study revealed that the aqueous BJ extract was effective in inhibiting the proliferation of NSCLC cells H1975^[22]^. To raise lung cancer tumorspheres, the adherent cells were cultured in a serum-free medium containing various growth factors. Under ultra-low attached environments, the residual cells formed suspension clusters. Growing in anchorage-independent conditions for 7 days, the distinctive bodies showing three-dimensional and well-rounded tumorsphere structures were gradually ameliorated by increasing BJ extract concentrations. In soft agar assay, the increased BJ concentrations progressively ameliorated H1975 spheroid clone numbers after 28 days [Figure 2A]. The concentration that inhibited 50% of spheroid colony-forming capacity was determined as 10 mg BJ/mL [Figure 2B].

### The aqueous BJ extract diminished stem cell profiles and enhanced apoptosis characteristics of H1975 spheroids under cultured conditions

The developed spheroids suspended in serum-free medium were treated with 5, 10, and 15 mg BJ/mL, respectively, of BJ extract for 12 h and collected. Western blot analysis of the protein lysates showed that BJ reduced EGFR, decreased phosphorylated EGFR^Y1068^, diminished Akt expression, and impaired Akt^S473 ^phosphorylation [Figure 2C]. The reduced Nanog and CD133 intensities of the spheroid lysates meant stemness attenuation as BJ concentrations were increased, whereas the intensities of cell membrane stem cell marker aldehyde dehydrogenase 1A1 (ALDH1A1) remained unchanged. Moreover, the cleavage of both poly(adenosine diphosphate ribose) polymerase (PARP) and procaspase-3 together with the appearance of active caspase 3 suggested that BJ extract induced apoptotic cell death in H1975 tumorspheres under cultured conditions.

### Gavage feeding of nude mice with BJ reduced the growth of xenograft tumors with inoculated spheroids

**Figure 3:**
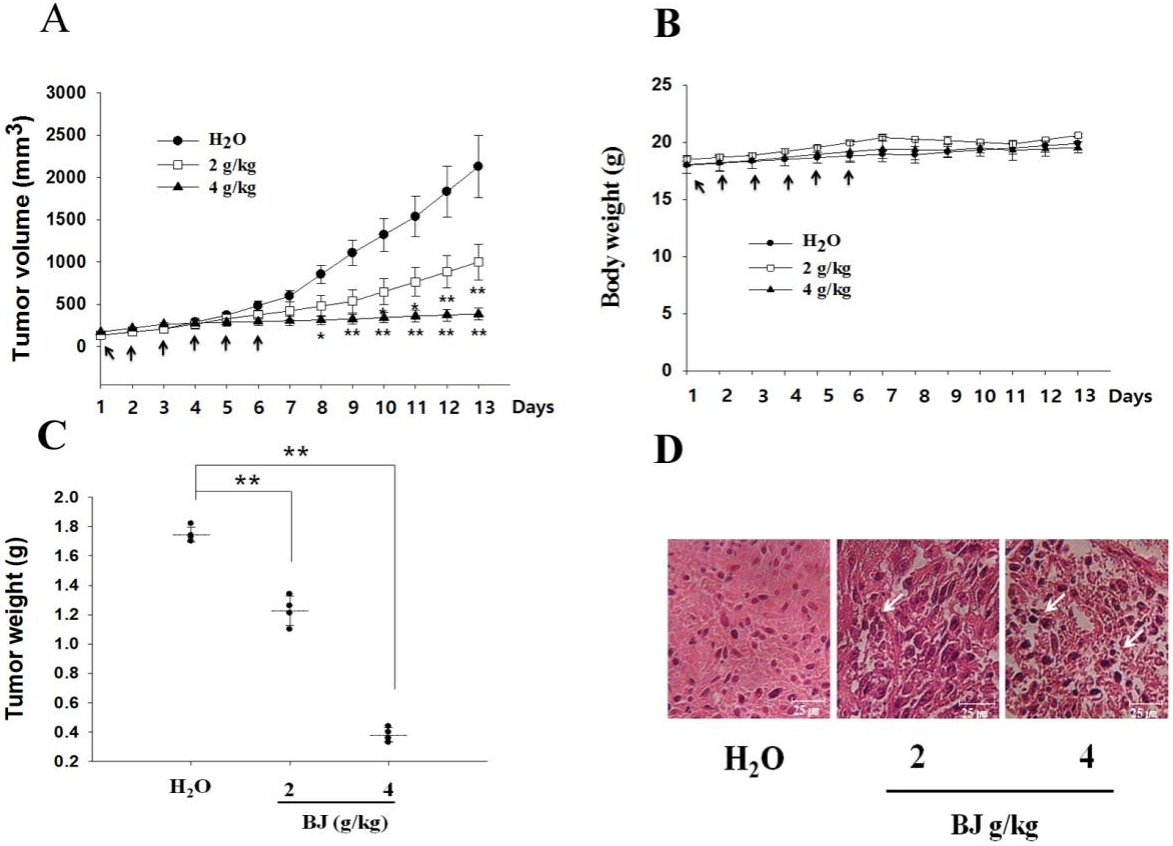
Oral administration of BJ reduced xenograft spheroid tumor growth. (A) BJ extract suppressed the growth of xenograft tumors. Nude mice with established xenograft spheroid tumors of H1975 cells as described in “Methods” were administered orally with 2 and 4 g of BJ/kg daily for 6 consecutive days (arrow). Each group contained four mice. The graph represents tumor growth variation (y-axis) from the start of feeding (x-axis). **P < 0.01 indicates a significant difference in the measured tumor volumes between mice fed with 2 and 4 g BJ/kg and those with water from three individual experiments with four mice in each group. (B) The bodyweight of nude mice. No significant differences of the average body weight in mice with established xenograft tumors orally administered with water and BJ extract daily for 6 consecutive days (arrow) were found. The graph represents variations of mice weight (y-axis) from the start of feeding in days (x-axis). (C) Resected tumor weight reduction. Tumor weights of the xenograft tumors were measured in mice with gavage feeding of BJ in comparison with those of water. The horizontal bars represent mean values of tumor mass as collected following different treatments. **P < 0.01 indicates significant weight difference between mice fed with 2 and 4 g of BJ/kg and those with water. The graph is representative of three independent experiments. (D) Hematoxylin and eosin (HE) staining. The paraffin-embedded spheroid tumors as treated with BJ (2 and 4 g/kg) and water-fed mice control were dissected, stained with HE, and analyzed by confocal microscopy (scale bar, 25 μm). The white arrow signifies apoptotic body location.

Xenograft tumors in nude mice appeared 14 days after inoculating each mouse with 8 × 10^4 ^spheroids as enriched from H1975 cells. Mice that received 2 and 4 g of BJ/kg showed steady relief of tumor burden compared with those fed with water alone [Figure 3A]. There was no apparent bodyweight loss in nude mice orally administered with aqueous BJ extract and water control [Figure 3B]. The increased BJ concentrations reduced the weight of dissected tumors [Figure 3C]. Tissues collected from tumors in mice fed with BJ displayed emerging apoptotic features in the histological images stained with hematoxylin and eosin. The apparent cytoplasm contraction, pyknosis, and interstitial space enlargement in tumor cells were increasingly visible in BJ-fed mice that differed from water control animals, in which the tumor cells were visibly viable [Figure 3D].

### The increased apoptosis and reduced PCNA of the implanted tumors in mice with administrated BJ

**Figure 4:**
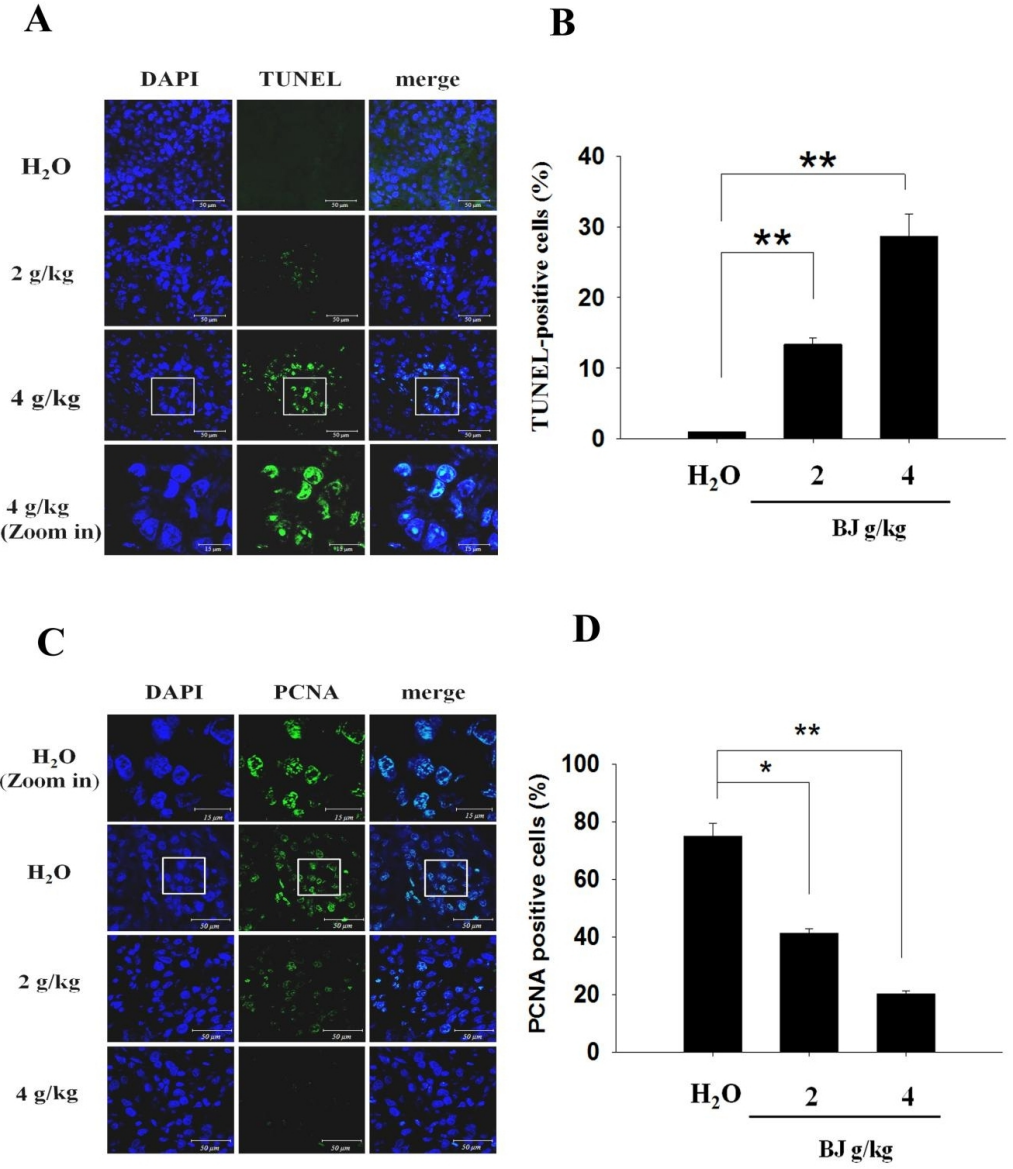
Spheroid tumor growth suppression. (A) Dose-dependent increase of TUNEL staining. The resected tumors in mice fed with water, 2 and 4 g of BJ/kg were frozen, resected, fixed, and subjected to TUNEL experiment for confocal microscopy analysis as described in “Methods”. The apoptotic bodies (green) were counter-stained with DAPI (blue) and visualized (scale bar, 50 μm). The image in the rectangular inset of tumor sections in mice treated with 4 g of BJ/kg is zoomed in at the bottom of the panel (scale bar, 15 μm). (B) BJ increased nucleus TUNEL-positive intensities. The numbers of fluorescent TUNEL-positive cells in each field containing 100 cells as marked by DAPI staining were counted. The numbers at various BJ concentrations were the average of at least three different fields. The data were expressed as mean averages of three individual experiments (**P < 0.01). (C) Dose-dependent decrease of stained nucleus mitotic index PCNA. The tumor sections were incubated with rabbit antibody against PCNA followed by FITC-conjugated secondary antibody. The slides with PCNA fluorescence (green) were counterstained with DAPI (blue) before being analyzed confocal microscopy (scale bar, 50 μm). The images in the rectangular inset of tumor sections in mice treated with water are zoomed in at the top of the panel (scale bar, 15 μm). (D) BJ decreased nucleus PCNA signals. The number of fluorescent nucleus PCNA-positive cells in each field containing 100 cells as marked by DAPI staining was counted. The numbers of various BJ concentrations were the averages of at least three different fields. The data were expressed as mean averages of three individual experiments (*P < 0.05, **P < 0.01).

The increasing TUNEL fluorescence in tissue sections from mice fed with BJ extract from 2 to 4 g/kg indicated the onset of apoptosis [Figure 4A]. The accentuated TUNEL fluorescence intensities meant elevated apoptotic death in tumors of mice receiving increasing extract concentrations in contrast with groups fed with water alone [Figure 4B]. Furthermore, the suppressed tumor growth in groups receiving extract showed diminished fluorescent mitotic index proliferating cell nuclear antigen (PCNA) [Figure 4C]. The distinct reduction of nuclear PCNA staining in the resected specimen demonstrated that BJ-induced apoptosis alleviated growth of xenograft tumors established by the inoculated spheroids [Figure 4D].

### The regressed fluorescent EGFR, pEGFR^Y1068^ and stemness markers in lung spheroid tumors

**Figure 5:**
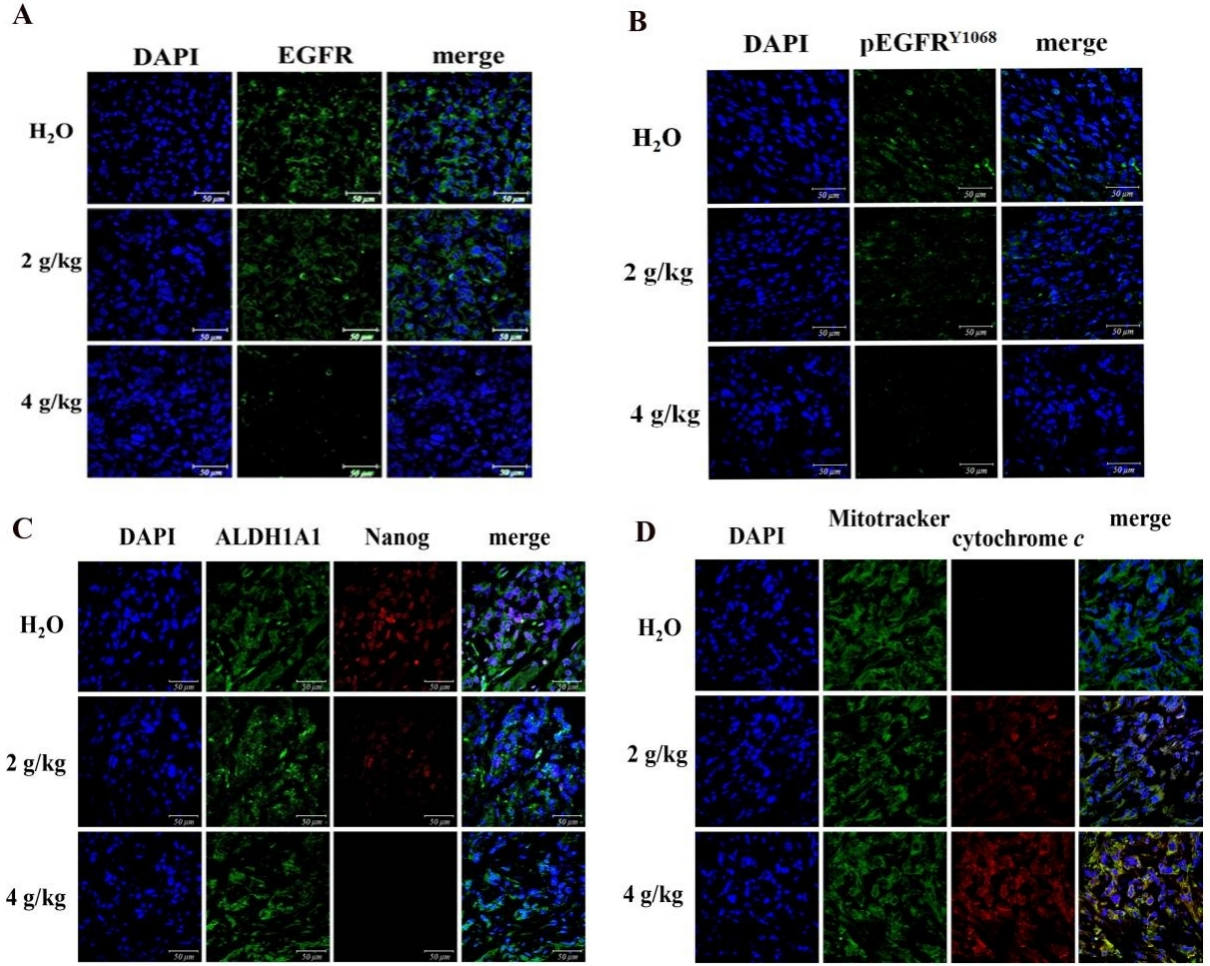
Fluorescence examination of the resected spheroid tumors. (A) Images of immuno-fluorescent EGFR. H1975 spheroid tumor sections from mice fed with 2 and 4 g/kg of BJ and water control were incubated with EGFR antibody (green) followed by FITC-conjugated secondary antibody treatment before being counter-stained with DAPI (blue) (scale bar, 50 μm). (B) Images of immuno-fluorescent pEGFR^Y1068^ H1975 spheroid tumor sections from mice fed with 2 and 4 g/kg of BJ and water control were incubated with pEGFR^Y1068^ antibody (green) followed by FITC-conjugated secondary antibody treatment before being counter-stained with DAPI (blue) (scale bar, 50 μm). (C) Images of immuno-fluorescent ALDH1A1 and Nanog H1975 spheroid tumor sections from mice fed with 2 and 4 of g/kg of BJ and water control were incubated with ALDH1A1 antibody (green) followed by FITC-conjugated secondary antibody. The Nanog antibody (red) incubation was followed by TRITC-conjugated secondary antibody before being counter-stained with DAPI (blue) and merged (scale bar, 50 μm). (D) Release of mitochondrial cytochrome *c* in spheroid tumors. The dissected specimens of H1975 spheroid tumors as treated with BJ (2 and 4 g/kg) and the water control were fixed and incubated with antibody against cytochrome *c* followed by staining with secondary antibody conjugated with TRITC (red). The slides were counter-stained with Mitotracker (green) and DAPI (blue) before being analyzed by confocal microscopy. The merged images from confocal microscopy with red cytochrome *c* and green mitochondria signify the appearance of coalescence (yellow), while blue indicates the nucleus (scale bar, 50 μm).

Administration of BJ diminished the intensive fluorescent EGFR [Figure 5A] and phosphorylated EGFR^Y1068 ^[Figure 5B] in tumor dissections, as indicated by antibody detection. In addition, BJ decreased fluorescent self-renewal signals including nucleus transcription factor Nanog that regulates the pluripotent inner cell mass during embryonic development^[23]^. In contrast, the cell surface stem cell indicator ALDH1A1 remained intact in the dissected H1975 spheroid tumor tissues within the concentration ranges [Figure 5C]. The emergent fluorescent puncta composed of coalesced green Mitotracker and the released red cytochrome *c* from mitochondria were clearly visible in tumor section images. The results suggest the emergent apoptotic death of H1975 spheroid tumor cells in mice receiving BJ extract [Figure 5D].

### The relapsed stem cell signatures accompanied with drug resistance marker in spheroid tumors

**Figure 6:**
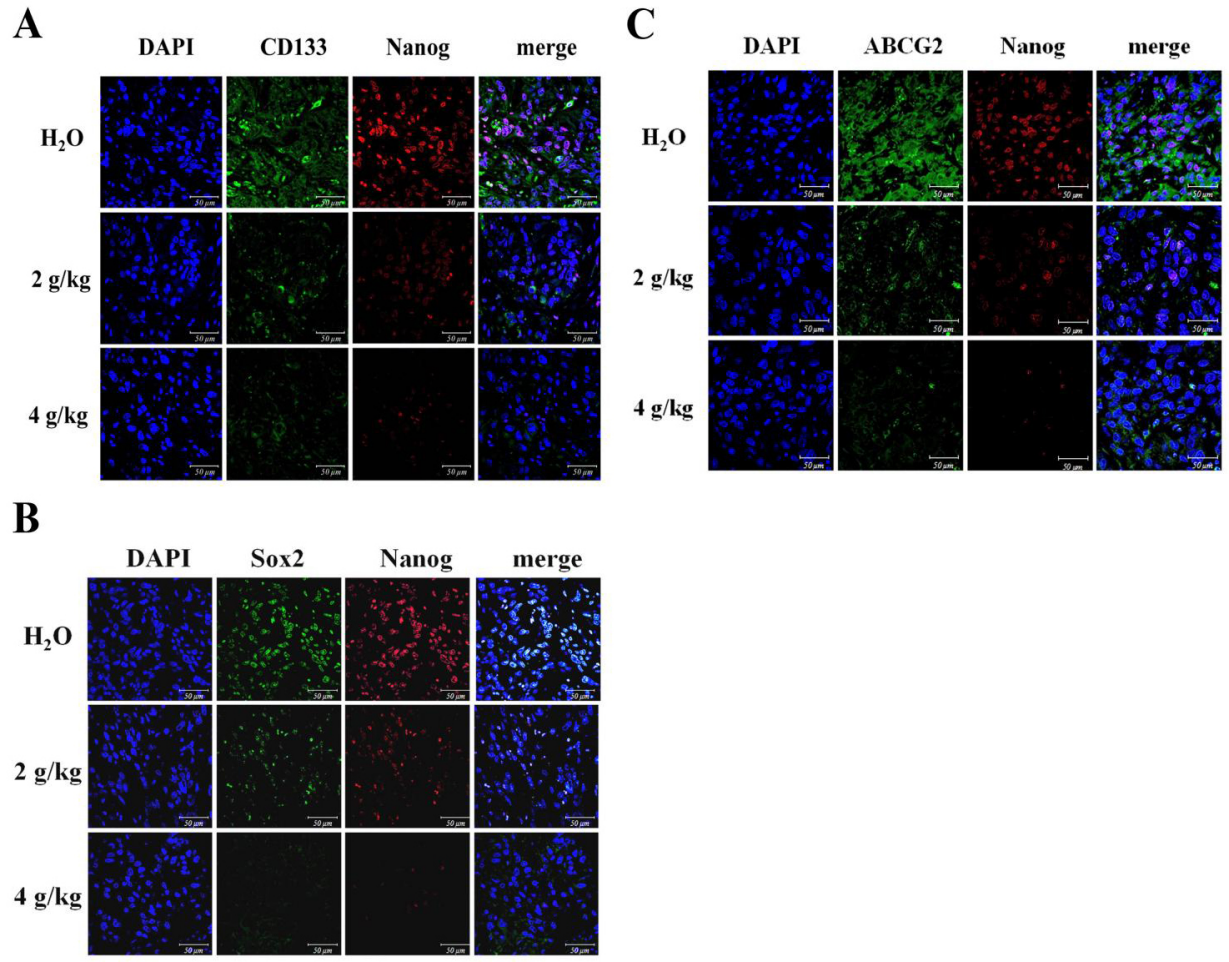
Fluorescent images of the resected tumor tissues: (A) the immuno-fluorescent intensities of CD133 (green) and Nanog (red); (B) the immuno-fluorescent Sox2 (green) and Nanog (red) images; and (C) the immuno-fluorescent ABCG2 (green) and Nanog (red) images. The spheroid tumor sections from mice fed with 2 and 4 g/kg of BJ and water control were incubated with primary antibodies followed by FITC- or TRITC-conjugated secondary antibodies, before being counter-stained with DAPI (blue)*.* Images taken from confocal microscopy were merged (scale bar, 50 μm).

The embryonic stem cell transcription factors Sox2 and Nanog are crucial in maintaining stemness features of tumorspheres originated from lung cancer^[24]^. The fluorescent stem cell markers including extracellular CD133 and nucleus Nanog were eradicated in spheroid tumors [Figure 6A]. The increased BJ concentrations reduced coalesced fluorescent nuclear stem cell markers Sox2 and Nanog [Figure 6B]. Cancer resistance protein ATP-binding cassette subfamily G member 2 (ABCG2) is presented primarily in stem cell membranes of lung cancer tumors^[2]^. The decreased dual staining of cell surface ABCG2 and nuclear Nanog signaled abolishment of drug resistance in H1975 sphere tumors following administration of BJ [Figure 6C].

### The attenuated EMT and c-Met in tumors of mice receiving BJ

**Figure 7:**
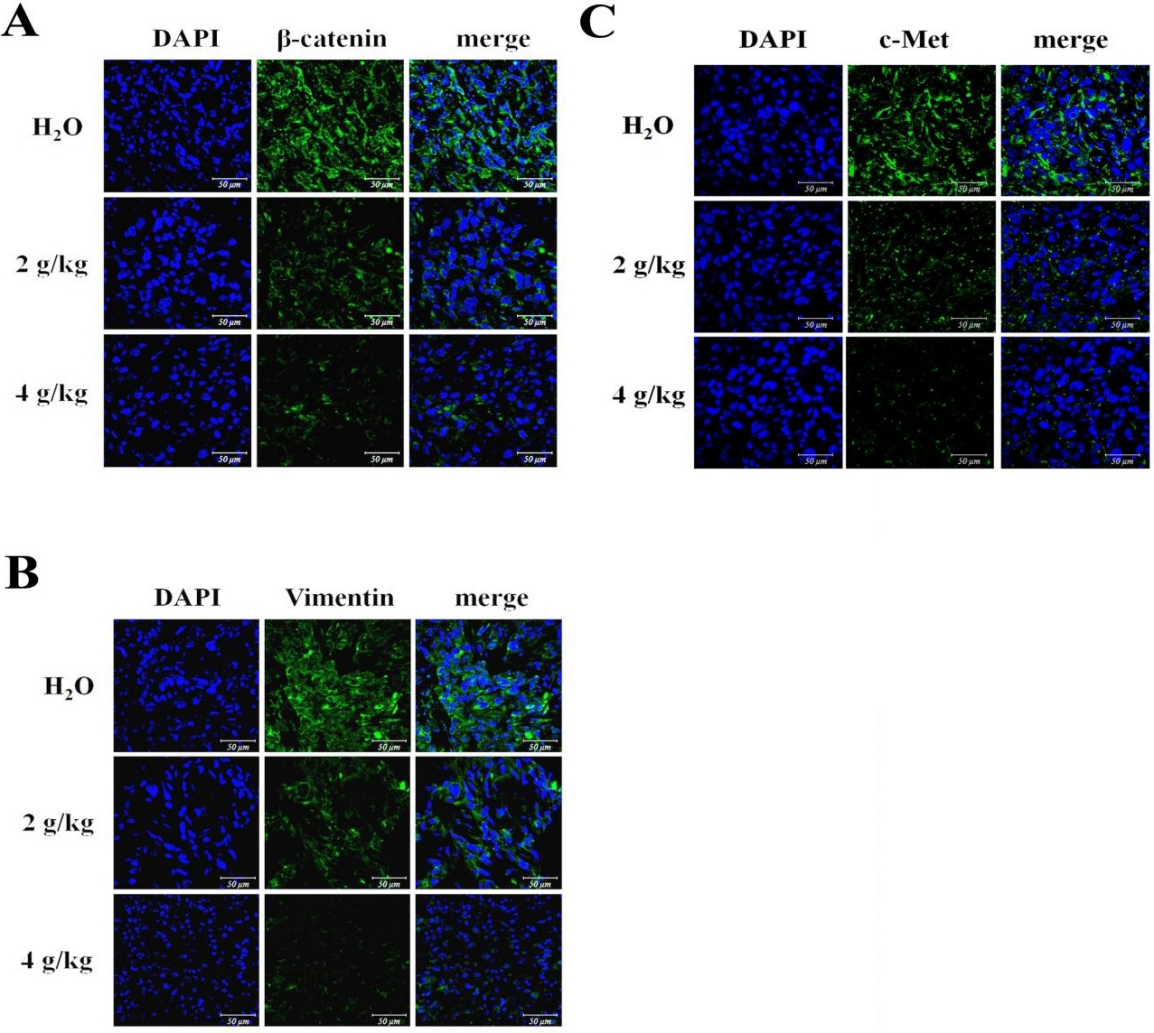
Fluorescent images of the resected tumor tissues: (A) the immuno-fluorescent β-catenin (green) images; (B) the immuno-fluorescent vimentin (green) images; and (C) the immuno-fluorescent c-Met (green) images. H1975 spheroid tumor sections from mice fed with 2 and 4 g/kg of BJ and water control were incubated with primary antibody followed by FITC- or TRITC-conjugated secondary antibody before being counter-stained with DAPI (blue). Images from confocal microscopy were taken and merged (scale bar, 50 μm).

NSCLC stem cells with T790M EGFR with intensive EMT are known to be insensitive to TKI treatment^[10]^. The acquired embryonic stem cell-like signature and the emerged EMT in tumorspheres are closely related^[24]^. The induced EMT characters in the emergent stem cell-like features is a key process by which cancer cells acquire invasive and metastatic phenotypes^[25,26]^. In CSC-implanted tumors from mice treated with increasing BJ extract concentrations, the intrinsic EMT markers were dissipated as indicated by the attenuated β-catenin [Figure 7A] and the reduced vimentin [Figure 7B] signals. 

Activation of c-Met-hepatocyte growth factor axis constitutes a major obstacle that contributes to gefitinib resistance^[27]^. *EGFR T790M* mutation and c-Met amplification are related to resistance to EGFR-TKI^[28]^. BJ was also shown effective at mitigating c-Met signaling as lung cancer spheroid tumors were gradually eliminated [Figure 7C]. 

## DISCUSSION

Human tumor cells endowed with self-renewal ability and stemness characteristics were tumorigenic and impermeable to treatment^[3]^. Tumorspheres from H1975 cells were developed as cluster suspension and cultured in serum-free medium supplemented with growth factors. The aqueous extract with identified *bruceolide* quassinoid mixture is effective against human lung cancer H1975 cells with double mutant EGFR^[22]^. In this study, the formation of the enriched tumorspheres was further proved susceptible to aqueous BJ extract. Inhibition of 50% of spheroid colony-forming capacity was determined at 10 mg/mL as a result of the induced apoptosis and the reduced stemness properties.

Orally administered BJ successfully eliminated H1975 spheroid tumor burden without affecting animal healthiness. Fluorescent analysis of the dissected tumor tissues showed that BJ extracts reduced fluorescent EGFR and its phosphorylated forms. The enriched stem cell signatures, including pluripotent stem cell markers CD133 and Sox2, drug resistance marker ABCG2, and transcriptional factor Nanog in H1975 spheroid tumors, were all reduced in tumors of mice administered with BJ extract. However, the surface marker specific for therapeutic resistance involving detoxification, ALDH1A1, remained intact in cultured spheres and in the established tumors at all experimental conditions. It is reasonably concluded that the established tumors were indeed from the enriched tumorspheres that can be progressively eliminated with increasing BJ extract concentrations. EGFR-mutant NSCLC cells equipped with ALDH1A1 activity are refractory to erlotinib but sensitive to active herbal ingredients^[29]^. Lung cancer cells with stem cell features are known to be insensitive to drug intervention^[30]^. More evidence suggests a linkage between undifferentiated stem cells and EMT expression^[31]^. Moreover, cancer cells with overexpressed EMT, intensive c-Met, and stem cell traits tend to be resistant to therapy^[32]^. The current study demonstrated that the growth of spheroid tumors with amplified drug resistance gene ABCG2 is suppressed by depleting stem-like markers and promoting apoptosis in stem-like cells bearing mutant EGFR.

The common anti-cancer therapies tend to retain residual subpopulations containing mostly malignant tumor progenitor cells with poor prognosis, robust resistance to therapy, and relentless recurrence^[3]^. Another study suggested that a combination of herbal medicine and gefitinib helped in gefitinib efficacy in drug-resistant NSCLC by reducing c-Met and EGFR interaction^[33]^. The previous report showed BJ extract suppressed the growth of liver cancer stem-like cells by attenuating EGFR expression^[20]^. The current findings underscore the significance of the extract composed of quassinoids, which not only repressed EGFR expression but also alleviated tumor growth of lung cancer spheroids by reducing drug resistance and activating apoptosis. The decreased stem cell markers of EMT and c-Met as well as overcoming drug resistance signaled the opportunities of the BJ extract for therapy of lung cancer harboring mutated EGFR.

In conclusion, BJ extract targeted NSCLC spheroid from recalcitrant cells with double mutant EGFR. This study demonstrated the potency of aqueous BJ extract in eradicating spheroid tumors as established by NSCLC cancer tumorspheres. The study also suggested the effectiveness of oral administration of BJ extract for clinical application. The decreased stemness property, drug resistance, and EMT of the enriched spheroids lead to the final apoptotic death of tumorspheres both in culture and in animal model. The findings also underscore that the conventional herbal medicine and its active ingredients are promising for potential translational application to eliminate lung cancer resistance to conventional therapy.

## DECLARATIONS

### Copyright

© The Author(s) 2021.
